# Implementing a specialist paediatric clinical pharmacology service in a UK children's hospital

**DOI:** 10.1111/bcp.14944

**Published:** 2021-08-22

**Authors:** Daniel B. Hawcutt, Naomi Warner, Elaine Kenyon, Christine Murray, Julia Taylor, James Moss, Stephen McWilliam, Will Weston, Nicki Murdock

**Affiliations:** ^1^ Women's and Children's Health, Institute of Life Course and Medical Sciences University of Liverpool Liverpool UK; ^2^ NIHR Alder Hey Clinical Research Facility Alder Hey Children's Hospital Liverpool UK; ^3^ Alder Hey Children's Hospital Liverpool UK

**Keywords:** deprescribing, drug optimization, paediatric pharmacology, paediatrics, pharmacy

## Abstract

**Aims:**

Royal College of Paediatrics and Child Health subspecialist training in Paediatric Clinical Pharmacology and Therapeutics has been delivered in the UK for 20 years, but no specialist clinical services have been set up previously.

**Methods:**

Prospective audit and service evaluation of paediatric clinical pharmacology service pilot phase and dedicated service at a UK children's hospital.

**Results:**

Pilot scheme (May–October 2019), then weekly service (established June 2020). Service covers the High Dependency Unit, and inpatients with polypharmacy. The pilot demonstrated high levels of acceptance, with 89% of suggested medication changes agreed by lead clinical team, and success, with 97.5% of suggested changes continued until discharge/pilot completion. Economic analysis estimated direct annualised cost savings on medications of up to £10 000. After 20 ward rounds of the established service, 270 potential medication changes were identified, 213 were carried out (78.9%). The most common were deprescribing (*n =* 143), prescribing (*n =* 47) and dose adjustment (*n =* 8). Seventy‐five different medications were deprescribed, most commonly chloral hydrate (*n =* 12), Lactulose, ibuprofen, Bio‐Kult and sodium alginate (all *n =* 4). The percentage of inpatients prescribed ≥10 medications decreased from 38.5 to 32.1%, while the subset prescribed ≥20 medications decreased from 11.0 to 5.67%. The mean number of medicines prescribed decreased from 9.0 to 8.0, while the median was unchanged at 7. Annual Yellow Card reports of suspected adverse drug reactions more than doubled (*n =* 66).

**Conclusion:**

A UK model for subspecialist paediatric clinical pharmacology service delivery has demonstrated a positive clinical impact and could be replicated at other UK secondary/tertiary children's hospitals.

**What is already known about this subject**

- Paediatric clinical pharmacology and therapeutics subspeciality training has been available via the Royal College of Paediatrics and Child Health for >20 years.
- Consultants in this subspecialty have undertaken academic and general paediatric roles, but there have not been any established subspecialist services to date in the UK.
- Internationally, there are well established models of service delivery for paediatric clinical pharmacology.

**What this study adds**

- A paediatric clinical pharmacology service within a UK secondary/tertiary/quaternary children's hospital is accepted by clinical colleagues, and the changes in medications are sustained.
- Deprescribing of medications in children is the most commonly required action on a paediatric clinical pharmacology ward round, and this impacts on the trust rates of polypharmacy.
- At a trust level, paediatric clinical pharmacology can lead and/or support a range of medication related groups, from statutory committees such as drugs and therapeutics to specialist areas (prescribing of cannabis‐containing products, valproate in female children with epilepsy).

## 1 INTRODUCTION

Clinical Pharmacology is a distinct medical specialty that examines all aspects of the relationship between drugs and humans. In the National Health Service (NHS), it is the only medical specialty focusing on the safe, effective and cost‐effective use of medicines.^1^

Within adult medicine, clinical pharmacology has established how it contributes to NHS clinical service delivery, academic research, and undergraduate/postgraduate teaching.^2^ The Royal College of Paediatrics and Child Health (RCPCH) in the UK has developed and delivers an accredited subspecialist training scheme in paediatric clinical pharmacology and therapeutics.^3^, ^4^ The areas covered by this training, as well as roles for paediatric clinical pharmacologists in academia and teaching have been developed.^5^ However, unlike other countries, a clinical service has not been developed in the UK, with subspecialists in the UK contributing clinically via general paediatric consultant posts or in specialist positions (e.g. National Poisons Centre). Across Europe and in North America, centres of excellence exist where paediatric clinical pharmacology services are well established and contribute to patient care directly.^6^, ^7^, ^8^

Children and young people (CYP) deserve medical treatments that are of the same quality and safety as adults. Within paediatrics, despite formularies such as the British National Formulary for Children, there is evidence that prescriptions for similar conditions in CYP in different healthcare settings or geographical areas vary considerably, indicating that there are still considerable improvements to be made in implementing rational prescribing in the UK.^9^ In addition, the number of medications prescribed is increasing, such that 8% of all CYP take 2 or more medicines.^10^, ^11^ As treatments improve, the number of CYP surviving with complex conditions is also increasing,^12^ and these CYP have increased likelihood of problematic polypharmacy. The use of unlicensed and off label medications can be necessary in paediatric practice.^13^ New medications, and new indications for older medicines, require scrutiny at trust level on drugs and therapeutics committees. Adverse drug reactions (ADRs) are responsible for 3% of all paediatric admissions, and complicate 15% of inpatient stays,^14^, ^15^ but reporting to spontaneous reporting schemes to identify these suspected harms remains low.^16^, ^17^ There are initiatives and new technologies (e.g. deprescribing and pharmacogenomics) related to children's medicines that will require evaluation, and if appropriate, integration into NHS services. There is therefore a need for specialist paediatric clinicians whose focus is on the safe and effective use of medicines.

Pharmacists are another key group of healthcare professionals who contribute to the safe and effective use of medications in CYP, and there are many examples of improvements led and/or delivered by clinical pharmacists including identifying and preventing medication errors, identifying and reporting ADRs, and medicines reconciliation.^18^, ^19^, ^20^, ^21^ Recently, pharmacists in paediatrics have also participated and led *druggles*, a prescribing‐ or drugs‐themed huddle on paediatric wards.^22^, ^23^, ^24^ Additional services by paediatric clinical pharmacologists need to aware of these schemes, and ensure the work adds value rather than repeating existing work.

The aim of this work is to describe the clinical pharmacology service pioneered at a secondary/tertiary/quaternary children's hospital in England, and present prospective audit and quality improvement data related to the services activities.

## 2 METHODS

A senior lecturer with RCPCH‐accredited training in paediatric clinical pharmacology was employed at University of Liverpool, with honorary contract as a consultant paediatrician at Alder Hey Children's Hospital in 2013.

All activity described was undertaken at Alder Hey Children's Hospital NHS Trust, a standalone children's hospital based in Liverpool in the UK. It provides secondary, tertiary and quaternary paediatric healthcare services to children in Merseyside and beyond, with 270 inpatient beds, including 48 critical care beds for patients in intensive care unit, high dependency unit (HDU) and burns. An audit was registered with the hospital audit department (registration number 6018).

The Alder Hey pharmacy has previously developed a drug review round (druggle) on the HDU (lead clinician J.T.), where patients on this unit had their current medication checked by a pharmacist and specialist nurse. The HDU in Alder Hey part of the critical care unit, with 15 inpatient beds. The HDU manages medical and surgical CYP who correspond to Paediatric Intensive Care Society Level 1 and 2 (basic and intermediate) critical care,^25^ including noninvasive and long‐term ventilation via tracheostomy, arrythmias, severe asthma, diabetic ketoacidosis, reduced level of consciousness, status epilepticus and inotrope support.

A pilot phase was undertaken, where a single programmed activity (PA) of subspecialist consultant time was allocated each fortnight for a drug optimization ward round, and other pharmacology related activities. This ran from 2 May 2019 until 31 October 2019.

Following a business case submission, a dedicated clinical service was established on 11th June 2020, delivering 1 PA of consultant time per week. This was re‐allocation of a pre‐existing consultant resource from acute general paediatric admissions.

The clinical ward round was for both the pilot phase and clinical service were aligned with the existing druggle initiative on the HDU to ensure advanced nurse practitioner and specialist pharmacy input for the HDU patients. In addition, following the establishment of the clinical service, when available the consultant covering HDU also attends to provide additional clinical information.

Patients in other parts of the hospital were reviewed by the pharmacology team only. Patients not on HDU were prioritised by the number of active prescriptions on the trust electronic prescribing system, or by referral from clinical teams. The Trust IT department provided the pharmacology team with a report on PowerBI (Microsoft Corporation) showing live inpatient prescription data including location of patient, number and type of medications. Prospective audit of the pilot and subsequent service was registered with the trust audit department (Audit registration number 6165).

## 3 RESULTS

### 3.1 Drug optimization ward round

#### 3.1.1 Pilot phase

During the pilot phase, a total of 11 ward rounds were undertaken in the 26‐week pilot (expected number 13). Although attempts were made to reschedule, delivery was limited by participation in the acute paediatric rota.

During the pilot phase, the ward round suggested 95 changes to medications, including 66 medications be deprescribed. The number of patients seen per ward round increased across the time frame of the pilot; over the first 3 ward rounds, only 10 medicine changes were suggested in total (3.3 per ward round), but during the final 3 of the pilot, 35 changes were suggested (11.6 per ward round). A high level of clinical acceptance was noted, with 89% of suggested changes to medications agreed by lead clinical teams. Reasons for not implementing changes included lead clinical team having additional clinical information that made suggested change to medication unsuitable for that patient and proximity to discharge (do not want to “rock the boat”). Review of medications changes also showed a very high success, with 97.5% of suggested medicine changes were continued until discharge/completion of pilot scheme.

Using the trust's purchase costs for medications, not list price, the annualised direct savings from deprescribing were calculated. These took into account the individual patients length of time until discharge, that it was a mature ward round process (achieving 11.6 medication changes per ward round), and that it was undertaken weekly (45 times per year). The direct cost savings were calculated at £5726.61–10 027.80 per PA of consultant time. The range is present as there are a medications taken *as required* that could have a variable number of doses administered.

#### 3.1.2 Established service

The paediatric pharmacology service commenced in June 2020. The full list of roles and responsibilities included in the allocated time are shown in Table 1. A typical ward round consists of review of every patient on HDU (19 beds), followed by review of inpatients with polypharmacy on any ward in the hospital. Although remote review was possible using the PowerBI live report, and the e‐prescribing system, a personal review was undertaken to facilitate discussion with nursing staff, the patients and parents.

**TABLE 1:** Clinical activities associated with specialist paediatric clinical pharmacology consultant role in a secondary/tertiary children's hospital in the UK

Clinical Activity	Summary of role	Time commitment
Ward round	Review of HDU and wards for patients with polypharmacy	Up to 4 h/wk
Drugs and therapeutics committee	Committee overseeing medication use in the trust	2 h/mo
Clinical development and evaluation group	Rapid review of new medicines for use in the trust (XX applications June 2020 to Feb 2021 inclusive)	2 h/mo
Trust special committees/groups related to medicines	Member of valproate and cannabis containing medicines groups	1 h/mo
RCPCH/NPPG joint standing committee on medicines	Supporting education, training and research, in the field of paediatric prescribing	3 h quarterly

HDU: high dependency unit; RCPCH: Royal College of Paediatrics and Child Health; NPPG: Neonatal and Paediatric Pharmacists Group

Clinical delivery of the ward round was weekly, and data from the 20 of the first 23 ward rounds are shown below, from inception of the ward round to 11 February 2021 (35 weeks). Audit data were not captured for 3 ward rounds. Ward rounds were not delivered on 12 occasions due to annual leave (*n =* 5), COVID‐related disruption (*n =* 4) and alternate paediatric pharmacology service delivery (*n =* 3).

In addition to the ward reviews, additional aspect of the pharmacology ward round that developed during the clinical service period were:

- Contributing to multidisciplinary team assessments of complex patients with polypharmacy where coordination of care between hospital(s) and community was required, with written plans being created for patients
- Establishing ways for patients to access new clinical services (e.g. pharmacogenomic testing for actionable polymorphisms) to improve the quality of care

During the ward rounds, 72 patients, with 48 different lead consultants, were identified as potentially benefitting from alterations to their prescribed medications. Patients reviewed but in whom changes were not considered appropriate were not recorded. From this cohort of patients, 270 possible medication changes were identified, of which 213 were carried out (78.9%), at an average of 10.6 actions per ward round. The most common medication changes are shown in Table 2. Prescriptions for 75 different medications were changed. One medication intervention was reversed afterwards by the team as the patient developed low magnesium levels, and the supplements were re‐started. The medications that were changed on 3 or more occasions are shown in Table 2. The medications most commonly deprescribed were chloral hydrate (*n =* 12), Lactulose (*n =* 4), ibuprofen (*n =* 4), Bio‐Kult (*n =* 4) and sodium alginate (Gaviscon; *n =* 4). Octenisan (an antimicrobial body wash used in critical care) was the most commonly prescribed (*n =* 20).

**TABLE 2:** Types of actions, and medications changed as part of the paediatric clinical pharmacology ward round

Drug Optimisation Action	Frequency
Deprescribed	143
Prescribed	47
Dose alteration	8
Seek more evidence	4
Home medication list updated	3
Multidisciplinary team meeting	2
Weaning plan determined	1
Adverse drug reaction report	1
Time altered	1
Yellow card submission	1
Other (e.g. plans contingent on success of initial deprescribing)	2
**Medications changed** *	**Frequency**
Octenisan	23
Chloral hydrate	13
Lactulose	11
Omeprazole	6
Ibuprofen	6
Biokult	5
Gaviscon	5
Promethazine	4
Frusemide	4
Dioralyte	4
Morphine	4
Naloxone	4
Movicol	3
Paracetamol	3
Dexamethasone	3
Sodium feredetate	3
Midazolam	3
Beclomethasone	3

* Only medications changed 3 or more times are included

**FIGURE 1:**
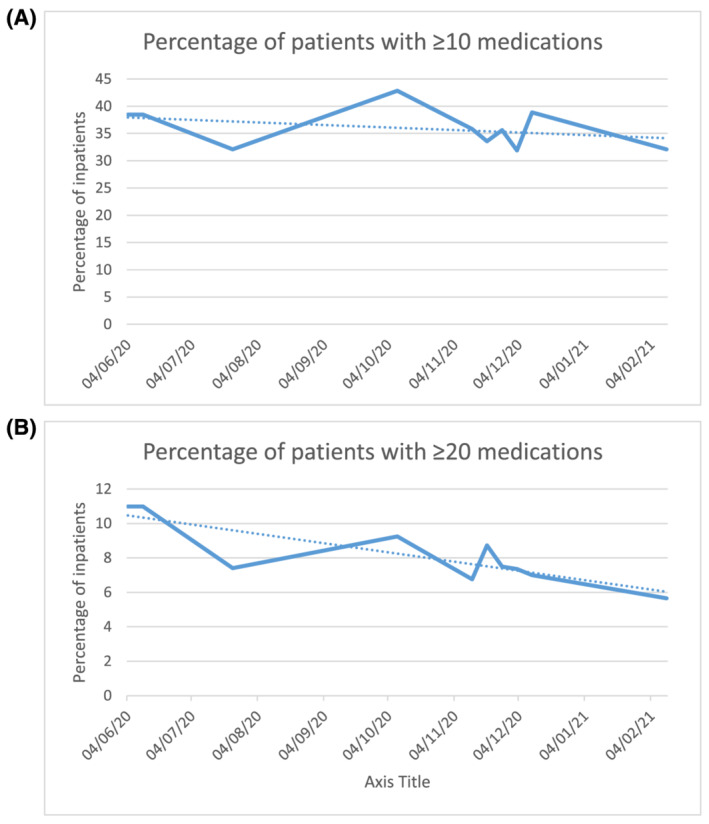
Percentage of inpatients prescribed. (A) ≥10 medications and (B) ≥20 medications

The percentage of children in the trust with polypharmacy has decreased during the time of the clinical service. At the start, 38.5% were prescribed ≥10 medications, while 11% of inpatients were prescribed ≥20 medications. At the time of analysis, the percentage had reduced to 32.1 and 5.7% respectively (Figure 1). The mean number of medicines prescribed decreased from 9.0 to 8.0, while the median was unchanged at 7.

#### 3.1.3 Trust level contribution

Drugs and Therapeutics Committee/Clinical Development and Evaluation Group.

The clinical pharmacology team contributed to the trust drugs and therapeutics committee as chair of both the drugs and therapeutics committee, and the subcommittee responsible for approval of new drugs and procedures (Clinical Development and Evaluation Group). In the period June 2019 to February 2021, 37 new submissions for new medications and/or procedures were reviewed. In addition, 27 urgent drug requests requiring rapid review (24–48 h) and response were received and responded to. Additionally, time has been dedicated to support trust committees and groups managing specific medication issues (e.g. sodium valproate, cannabis‐containing medications).

#### 3.1.4 Spontaneous reporting of suspected ADRs

**FIGURE 2:**
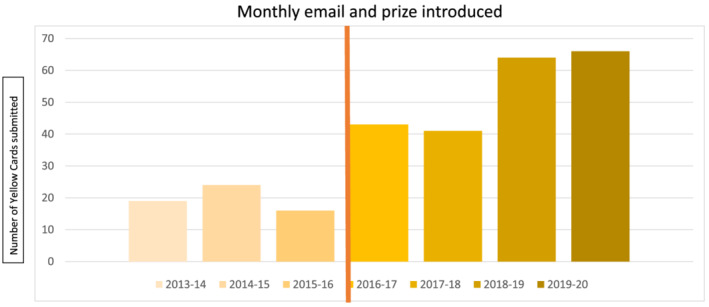
Annual number of reports of suspected adverse drug reactions to the Medicines and Healthcare Devices Regulatory Agency (MHRA) Yellow Card scheme from Hospital Trust

Reporting of suspected ADRs to the Medicines and Healthcare Devices Regulatory Agency has been championed by the clinical pharmacology service since the appointment of the honorary consultant. This has been through education of a range of trainees and staff of various healthcare professions, supporting medication safety officers, communication and engagement with medical and pharmacy colleagues, and, most successfully, through a monthly email and associated prize draw offering a coffee to a reporter chosen at random. This initiative has demonstrated a sustained improvement in the rate of reporting in the trust (Figure 2). ADRs reported on the pharmacology ward round, and captured through promotional activity and monthly emails are shown in Table 3. Of the 52 medications suspected overall, there were 46 different drugs, with none included on more than 3 reports, and only a single vaccine reaction noted. The types of suspected ADRs reported were not formally scored for severity, but included clinically significant events such as anaphylaxis, acute kidney injury, intracranial haemorrhage, and demyelinating illness.

**TABLE 3:** Suspected adverse drug reactions, and the medication suspected, identified by the pharmacology service and through monthly adverse drug reaction promotional activity

Suspected drug(s)	Suspected adverse drug reaction
5‐fluorouracil/dexamethasone	Bradycardia, requiring high dependency unit admission
Aciclovir	Acute kidney injury (AKI)
Ampicillin	Neutropenia
Atenolol	Stiffening & vacant episodes, constipation & sleeplessness
Azithromycin	Onychomadesis
Cefuroxime	Delayed hypersensitivity
Ciprofloxacin	Eosinophilia
Codeine and paracetamol (adult)	Chest pain
Cyclizine	Swollen eyes, rash
Cyclizine	Oculogyric crisis
Cyclopentolate eye drops	Allergic reaction
Daptomycin	Rash
Desflurane	Hepatotoxicity
Dexamethasone	Cataract
Domperidone	Perineal skin breakdown (secondary to loose stool)
Emicizumab	Pain on administration, reduced adherence, haemorrhage
Esketamine	Seizure
Flu vaccine	Haematuria
Gabapentin	Neuropsychiatric reaction
Gabapentin	Photodermatitis
Hyoscine patches	Nosebleeds
Ibuprofen	AKI grade 2
Ibuprofen	AKI
Imatinib	Intracranial bleed
Infliximab	Infusion reaction and respiratory distress
Infliximab	Demyelinating illness
Intravenous immunoglobulin	Aseptic meningitis
Ivacaftor/tezacaftor/elexacaftor.	Distal intestinal obstruction syndrome
Ledipasvir/Sofosbuvir.	Nausea and headache
Linezolid	Black tongue discolouration
Lumicaftor/ivacaftor	Dry eyes
Meningo B vaccine	Cerebrospinal fluid pleocytosis
Mepolizumab	Severe infection
Methylphenidate	Intracranial bleed
Metoclopramide	Allergic reaction
Montelukast	Muscle aches and cramps
Montelukast	Bed wetting
Morphine	Hyponatraemia
NItrazepam	Urinary frequency
Pamidronate	Rhabdomyolysis
Phenobarbitone	Respiratory depression
Phenytoin	Toxic epidermal necrolysis
Phenytoin	Elevated liver enzymes
Phenytoin/cefotaxime	Drug rash with eosinophilia and systemic symptoms syndrome
Piperacillin/tazobactam	Acute anaphylaxis
Piracetam	Reduced level of consciousness
Remdesevir	Black stool
Risperidone	Heart block
Sufadiazine/pyrimethamine	Renal calculi
Topiramate	Osteoporosis
Topiramate	Weight loss
Urografin	Distress during infusion
Vincristine	Mucositis requiring admission to hospital

## 4 DISCUSSION

The paediatric clinical pharmacology service is the first service of this kind in the UK and has been developed over several years. The service has demonstrated that it can be integrated into a secondary/tertiary/quaternary children's hospital. It has been reassuring to see the level of clinical acceptance of the service, with overwhelming support from medical and surgical consultants, as well as a wide range of other healthcare professionals, and it would not have been possible to deliver it without this. It has also been very positive to see that teams have been happy to review and change medications based on the services suggestions.

The service is now in discussion with the local Clinical Commissioning Groups (CCGs) to develop an outpatient referral service, which would facilitate the follow up of the CYP with problematic polypharmacy. This has the potential to increase the reach of the service, as well as prove extremely cost effective to the NHS as the prices paid for medications by a CCG are not the same as they are in a large trust where discounts may be negotiated (but are confidential). The annualised cost savings shown here are using the trust costs, so represent a lower estimate of the potential savings for CCGs if the service were expanded. They also do not include any potential indirect cost savings from reduced potential for ADRs, drug–drug interactions etc.

The RCPCH offers higher specialist training in paediatric clinical pharmacology and therapeutics, to develop the specialist consultants to deliver a service like this in equivalent centres if the clinical need is felt to exist. The current training scheme is full, but with only 2 places nationally it will take many years to significantly increase the number of trained individuals. This seems particularly important currently, given the national focus on rapid assimilation of research findings into clinical practice with regard to COVID‐19, and the associated therapeutic uncertainties, areas where clinical pharmacology can be particularly useful. In addition, trained pharmacologists can contribute beyond the clinical service described here, as highlighted in the recent British Pharmacological Society's response to the Department of Health and Social Care strategy *The Future of Clinical Research Delivery*.^26^

The service described worked alongside the existing HDU druggle. The 2 services worked well, with expertise of the advanced nurse specialists, specialist pharmacists, HDU consultants and Paediatric clinical pharmacology producing a large number of interventions. Importantly, the type of decisions made were felt to be different from the previous druggles round, and formal review of this is planned in future.

While the clinical service described may be transferrable to other large secondary/tertiary/quaternary children's hospitals, most children and young people are looked after in district general hospitals (DGH) where the paediatric department is 1 part of a larger institution. Therefore, while there is paediatric clinical pharmacology subspecialist training already, that can train specialist pharmacologists for roles in highly specialist children's hospitals, there is a gap currently. However, paediatric clinical pharmacology consultants nationally, working with the RCPCH, are developing a specialist training (SPIN) module for general paediatricians. This SPIN module is aimed at a DGH paediatrician, to ensure the needs of children with regards to medicines are fully considered in a DGH environment, and the aim is to have this training in place for 2022.

While the data presented here do appear very positive, we do acknowledge that due to the service being commenced in the middle of the COVID‐19 pandemic, the clinical situation overall, and the inpatients present in the hospital, may not be representative of a *normal* winter period in the north‐west of England. However, the service will continue to prospectively audit their intervention data to monitor changes in inpatient case mix, and track any improvements that are sustained. The delivery of this service has also thrown into sharp relief the lack of data supporting clinicians wishing to deprescribe medications in paediatrics. This has led to ongoing academic research about how to develop such guidance, and how to disseminate it effectively to where it is needed.

The suspected ADRs identified were from a wide range of drugs, and the pattern of medications differs markedly from both the overall Yellow Card Reports for children and neonates in the UK,^16^ but also those from CYP themselves,^27^ demonstrating the necessity of having multiple routes of reporting to capture the full range of possible ADRs.

## 5 CONCLUSION

A UK model for subspecialist paediatric clinical pharmacology service delivery has demonstrated a clinical impact at a patient and hospital level that could be replicated at other UK secondary/tertiary children's hospitals.

## DATA SHARING

Data available on request due to privacy/ethical restrictions.

## COMPETING INTERESTS

The authors have no conflicts of interest to declare.

## CONTRIBUTORS

Pharmacology ward round and audit conceived and undertaken, manuscript drafting (D.H.). Ward round and manuscript review/editing (N.W., E.K., C.M., J.M., S.M.). Druggle lead, ward round and manuscript review/editing (J.T.). Service development, manuscript review/editing (W.W., N.M.). Dr Hawcutt is the Principal Investigator on this manuscript.

## Data Availability

Data available on request due to privacy/ethical restrictions.
